# Ionic Conduction Mechanism and Design of Metal–Organic Framework Based Quasi-Solid-State Electrolytes

**DOI:** 10.1021/jacs.2c03710

**Published:** 2022-06-14

**Authors:** Tingzheng Hou, Wentao Xu, Xiaokun Pei, Lu Jiang, Omar M. Yaghi, Kristin A. Persson

**Affiliations:** † Department of Materials Science and Engineering, University of California, Berkeley, California 94720, United States; ‡ Energy Technologies Area, Lawrence Berkeley National Laboratory, Berkeley, California 94720, United States; § Department of Chemistry, University of California, Berkeley, California 94720, United States; ∥ Materials Sciences Division, Lawrence Berkeley National Laboratory, Berkeley, California 94720, United States; ⊥ The Molecular Foundry, Lawrence Berkeley National Laboratory, Berkeley, California 94720, United States

## Abstract

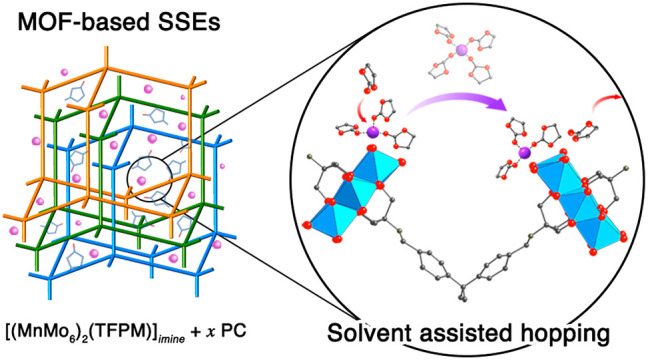

We report the theoretical and experimental investigation of two polyoxometalate-based metal–organic frameworks (MOFs), [(MnMo_6_)_2_(TFPM)]_*imine*_ and [(AlMo_6_)_2_(TFPM)]_*imine*_, as quasi-solid-state electrolytes. Classical molecular dynamics coupled with quantum chemistry and grand canonical Monte Carlo are utilized to model the corresponding diffusion and ionic conduction in the two materials. Using different approximate levels of ion diffusion behavior, the primary ionic conduction mechanism was identified as solvent-assisted hopping (>77%). Detailed static and dynamic solvation structures were obtained to interpret Li^+^ motion with high spatial and temporal resolution. A rationally designed noninterpenetrating MOF-688(one-fold) material is proposed to achieve 6–8 times better performance (1.6–1.7 mS cm^–1^) than the current state-of-the-art (0.19–0.35 mS cm^–1^).

Solid-state electrolytes with high mechanical strength and ionic conductivity are anticipated to revolutionize the energy storage industry.^1−5^ This is due to their significant contributions to improved safety, low-temperature performance, and volumetric energy density as compared to conventional liquid electrolytes. Recently, anionic metal–organic frameworks (MOFs) with superior ionic conductivity and Li^+^ transference numbers have opened a new avenue in the development of quasi-solid-state electrolytes (QSSEs).^6−11^ To immobilize anions on the backbone of these frameworks, one approach is to directly link negatively charged building blocks, whereby the lithium counterions are introduced as the only mobile species inside the material.^9^ For example, a three-fold interpenetrating anionic MOF (MOF-688) was synthesized from Anderson type polyoxometalate (POM) [N(C_4_H_9_)_4_]_3_[MnMo_6_O_18_{(OCH_2_)_3_CNH_2_}_2_] (MnMo_6_) and tetrakis(4-formylphenyl)methane (TFPM) building units through imine condensation.^12^ With propylene carbonate (PC) filling the pores, Li^+^-exchanged MOF-688 exhibited a high ionic conductivity of 4.0 × 10^–4^ S cm^–1^ and high Li^+^ transference number of 0.87 at 298 K.

In this new class of promising prototype QSSEs, it is important to understand the transport and conduction mechanisms, especially given the characterization challenges associated with transport measurements.^13−15^ Solid-state nuclear magnetic resonance characterizations have shown that Li^+^ transport in the framework channels involves complex interactions between cations, anions, and framework segments^16^ and may exhibit a change in conduction mechanism with varying temperature.^17^ Moreover, given the vast materials space that results from linking inorganic nodes and organic ligands,^18^ it is important to develop theoretical methods that can predict and optimize the transport properties of MOFs to support the experimental efforts.^9^ Yuan et al.^19^ computed the energetics of Li^+^ hopping between binding sites in Cu-MOF-74, which provides support for a hypnotized hopping mechanism of Li^+^ conduction.^8^ However, the detailed ionic transport mechanisms and how exactly the anionic species and solvent molecules cooperatively facilitate the Li^+^ diffusion are still unclear.^9^

Classical molecular dynamics (MD) simulations have shown excellent results in modeling the solvation and transport properties of liquid electrolytes^20,21^ as well as the diffusion and adsorption properties of MOFs,^22,23^ and have been considered as a promising tool to provide in-depth understanding of MOF-related QSSEs.^9^ In this contribution, by combining MD simulations with quantum chemistry and grand canonical Monte Carlo (GCMC), we identified solvent-assisted hopping as the dominant pathway for Li^+^ conduction in MOF-688 materials, revealing the critical role of the solvent in MOF-based QSSEs. This work constitutes the first theoretical model that accurately describes the ionic conduction mechanism of MOF-based QSSEs at an atomistic level, which is challenging to obtain from experimental results,^13,14^ and provides guidance for possible improvements.

A molecular simulation model, denoted as MOF-688(Mn), was created from X-ray single crystal structure of MOF-688, [(MnMo_6_)_2_(TFPM)]_*imine*_.^12^ In parallel, an isoreticular structure [(AlMo_6_)_2_(TFPM)]_*imine*_, termed MOF-688(Al), was synthesized and modeled by substituting Mn^3+^ with Al^3+^ to investigate the influence of the POM center metal ion on ionic conduction. Li^+^ counterions were introduced in both MOFs with a POM/Li^+^ ratio of 1:3 (Supporting Information (SI) Section 1). Hybrid MD and GCMC simulations^24^ were performed to equilibrate the content of PC solvent in the pores of MOFs. The obtained PC-infused structures with a POM/PC ratio of 16:170 and a Li^+^ concentration of 2.0 mol L^–1^ were then utilized to perform MD simulations (SI Section 3). By analyzing MD trajectories over a few tens of nanoseconds, three different types of Li^+^ motion were observed (Scheme 1): (1) Li^+^ hopping on the same POM cluster between the outmost oxygens (i.e., binding sites); (2) Li^+^ hopping between POM clusters; and (3) solvated Li^+^ diffusion, where Li^+^ is coordinated and separated only by PC that can freely diffuse in bulk solvent.

**Scheme 1:**
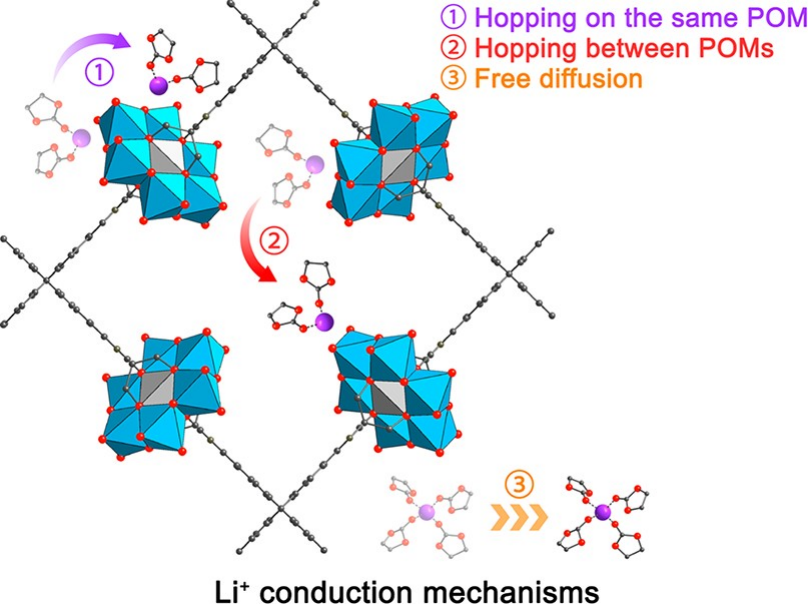
Three Proposed Conduction Mechanisms for MOF-688

To identify the ionic conduction mechanism of the two model materials, we calculated the ionic conductivity using different levels of approximation (SI Section 5): first using rigorous Green–Kubo (GK) relations which account for intermolecular transport correlations, second using the dilute approximation Nernst–Einstein equation, and third, using a single-mechanism hopping model. Comparing results of the three models enabled us to conclusively determine the dominant conduction mechanisms of both materials.

The calculated GK conductivity of MOF-688(Mn) at 298 K (Figure 1c) and as a function of temperature (Figure S12) agree well with the experimental trend, validating that the molecular model is suitable for the quantitative study of transport phenomena of MOF-based QSSEs. Moreover, MOF-688(Al) exhibits a slightly lower ionic conductivity than MOF-688(Mn), while the difference is within the error range. This observation indicates that changing the type of center metal ions is unlikely to significantly affect ionic conductivity. In addition, the discrepancy of the solvation structure between the two model materials is minor (Figure 1a,b, Figure S7) and attributed to a slightly weaker interaction between Li^+^ and MnMo_6_ (Table S2). It is further found that the self-diffusion coefficients of tethered and solvated Li^+^ are similar (Figure S10). Considering that only 7% Li^+^ are fully detached from the POMs in both MOFs and solvated (Figure 1a), the diffusion of solvated Li^+^ is excluded from the major conduction mechanism.

**Figure 1:**
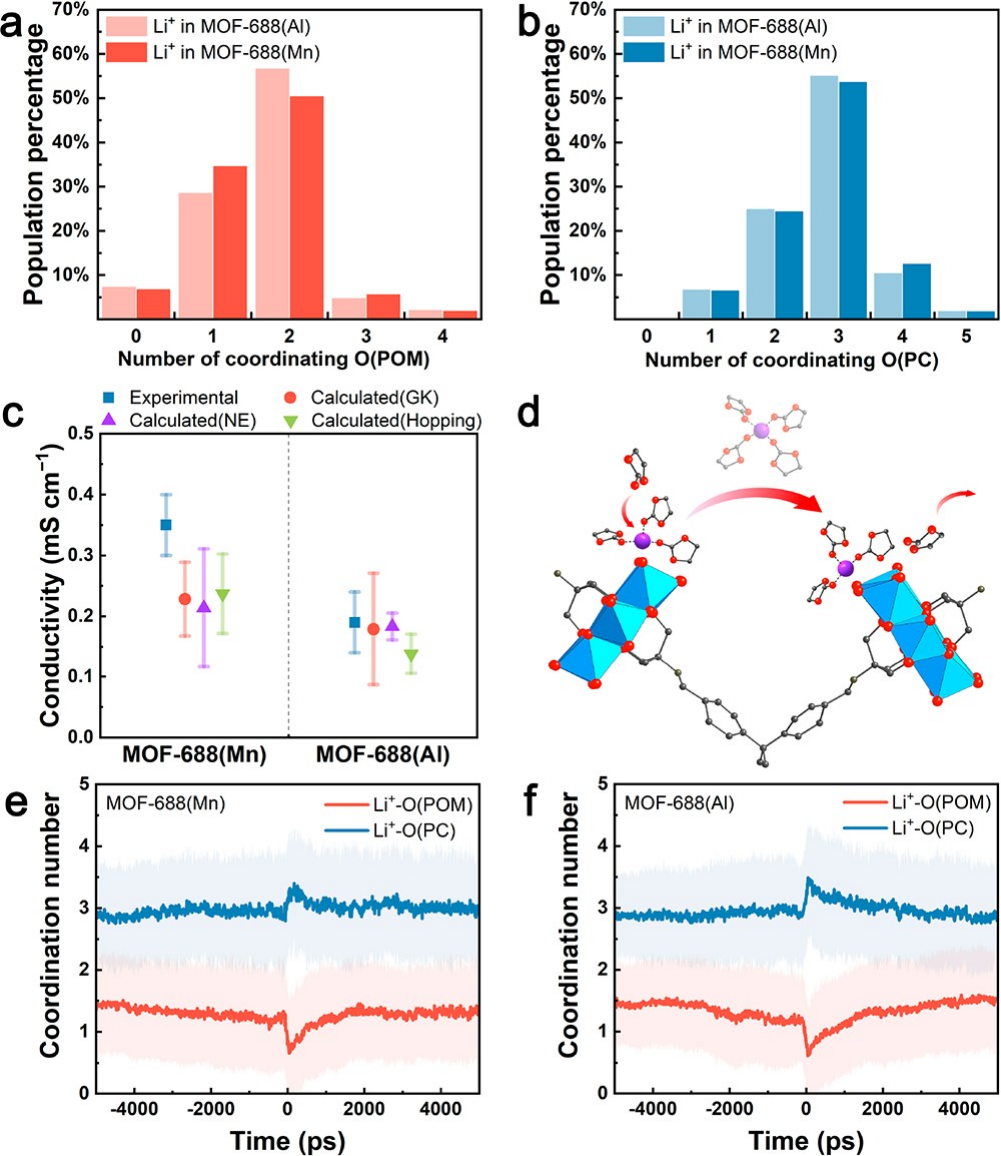
Conduction mechanism of MOF-688. The coordination number of (a) Li^+^-O(POM), and (b) Li^+^-O(PC) in MOF-688(Al) and MOF-688(Mn). (c) Ionic conductivities of MOF-688(Mn) and MOF-688(Al) from experimental measurements, and theoretical calculations using Green–Kubo relations (GK), Nernst–Einstein equation (NE), and simple hopping model (hopping). (d) Scheme of solvent-assisted hopping. The evolution of Li^+^-O(POM) and Li^+^-O(PC) coordination numbers before and after hopping in (e) MOF-688(Mn) and (f) MOF-688(Al). The light-colored area denotes the extent of standard deviation.

Next, we calculated the ionic conductivity assuming that the ionic conduction is mostly contributed by uncorrelated Li^+^ (self-)diffusion, and the intrinsically anionic frameworks are treated as fixed. With this assumption, the ionic conduction can be correlated to the self-diffusion coefficient of Li^+^ using the Nernst–Einstein (NE) equation. The computed NE conductivity is in fair agreement with the GK conductivity. Additionally, the concerted/correlated ion diffusion observed in all-solid-state electrolytes (e.g., NASICON^25,26^) is insignificant in the MOF-based QSSE, as analyzed using the Onsager transport theory (SI Section 5). Hence, the underlying assumption arguably holds that the conductivity is mostly contributed by Li^+^ self-diffusion, in agreement with the measured transference number (*t*_Li+_= 0.87).^12^

Finally, we calculated the ionic conductivity with a simple hopping model. The uncorrelated individual ion hopping can be described by a random-walk model.^27^ By incorporating the hopping diffusion coefficient into the NE equation, we obtain the hopping conductivity. While the calculation utilizes a simplified model, it yielded fair agreement with the other two models. On average, the hopping conductivity contributes to 100% and 77% of the GK conductivity of MOF-688(Mn) and MOF-688(Al), respectively, suggesting that Li^+^ hopping between POM clusters dominates Li^+^ diffusion.

The evolution of Li^+^ solvation sheath during Li^+^ hopping further reveals the solvent effect on the process. We observed discrete changes of average coordination numbers of both O(PC) and O(POM) before and after each hopping event (set to 0 ps) (Figure 1e,f). During hopping, the coordination number between Li^+^ and O(POM) decreases as Li^+^ no longer binds to previous binding sites and has not reestablished binding with another POM. Simultaneously, the average coordination number of O(PC) increases from 3 to 3.5 for both MOF-688(Mn) and MOF-688(Al). In the following 500–1000 ps after hopping, when Li^+^ is gradually tethered to the framework and the excess PC leaves the Li^+^ solvation sheath, the coordination numbers of O(PC) and O(POM) are restored to the bulk average. The direct involvement of the excess PC suggests that the primary mechanism of Li^+^ conduction is *solvent-assisted hopping* between POM clusters (Figure 1d), while the short residence time further indicates that the excess PC plays a temporary role rather than forming solvent-separated Li^+^ solvation structures that diffuse freely.

We find that the local charge distribution on the POM surface largely determines the interaction between Li^+^ and the framework. The electrostatic potential (ESP) surface of MnMo_6_ (Figure 2a,b) and AlMo_6_ (Figure S13) was calculated as a measure of the Coulombic interaction between POM and Li^+^. The ESP distribution of the two clusters is nearly identical, indicating that center metal ions with the same valency exhibit minor influence on the surface charge distribution of POM. The Li^+^ density distribution around MnMo_6_ (Figure 2c,d) coincides well with the ESP distribution, where the highest Li^+^ density is found on sites between two adjacent MoO_4_ moieties with the lowest electrostatic potential. This observation indicates that the electrostatic term is dominant in the interaction between Li^+^ and MnMo_6_, which further determines the most probable Li^+^ binding sites. In addition, Li^+^ can occasionally be monodentate-coordinated by one O as observed in the density plot.

**Figure 2:**
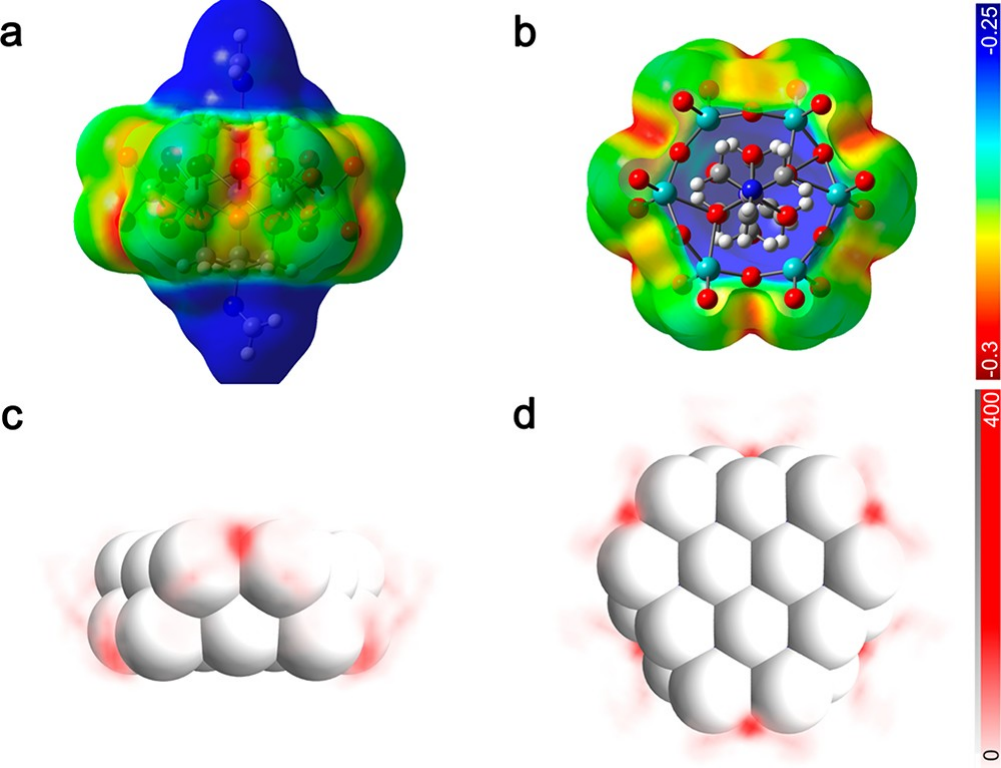
Charge and Li^+^ distribution on MnMo_6_ surface. (a) Front and (b) top view of the electrostatic potential (ESP) surface of MnMo_6_. Carbon, hydrogen, oxygen, nitrogen, and molybdenum atoms are represented by gray, white, red, blue, and cyan, respectively. The color bar shows the electrostatic potential in volts. (c) Front and (d) top view of the Li^+^ density plot on MnMo_6_ surface. White balls denote the oxygens in MnMo_6_. The color bar shows Li^+^ number count from 100,000 randomly sampled coordinates.

The revealed correlation between the charge distribution and Li^+^ distribution has important implications for the rational design of MOF-based QSSEs. Strongly localized charges that are immobilized on POM are expected to produce a tightly confined Li^+^ distribution. The overbinding between Li^+^ and the framework may lead to less favorable Li^+^ hopping with decreased mobility and ionic conductivity. Therefore, we surmise that more distributed local charges on POM are expected to facilitate weaker binding with Li^+^ and to enhance Li^+^ motion.^7^

The solvent is another important factor regulating Coulombic interaction. In MOF-based QSSEs, the solvent screens Li^+^ from the anionic frameworks, an aspect that results in less correlation between the Li^+^ motion and the framework.^28^ The apparent and effective Li^+^ concentrations in MOF-688(Mn) are calculated to be 2.0 and 2.6 mol L^–1^, respectively (SI Section 10).^29^ The substantial Li^+^/PC ratio (1:3.54) and corresponding Li^+^ concentration suggest a high ionic strength in the material. The low PC content is mainly due to the limited pore volume resulting from the interpenetrating frameworks (Figure 3a). Therefore, we propose that reducing the degree of interpenetration could be an effective approach to increase the pore volume and accordingly the amount of solvent in pores, thereby reducing the viscosity and ionic strength.

**Figure 3:**
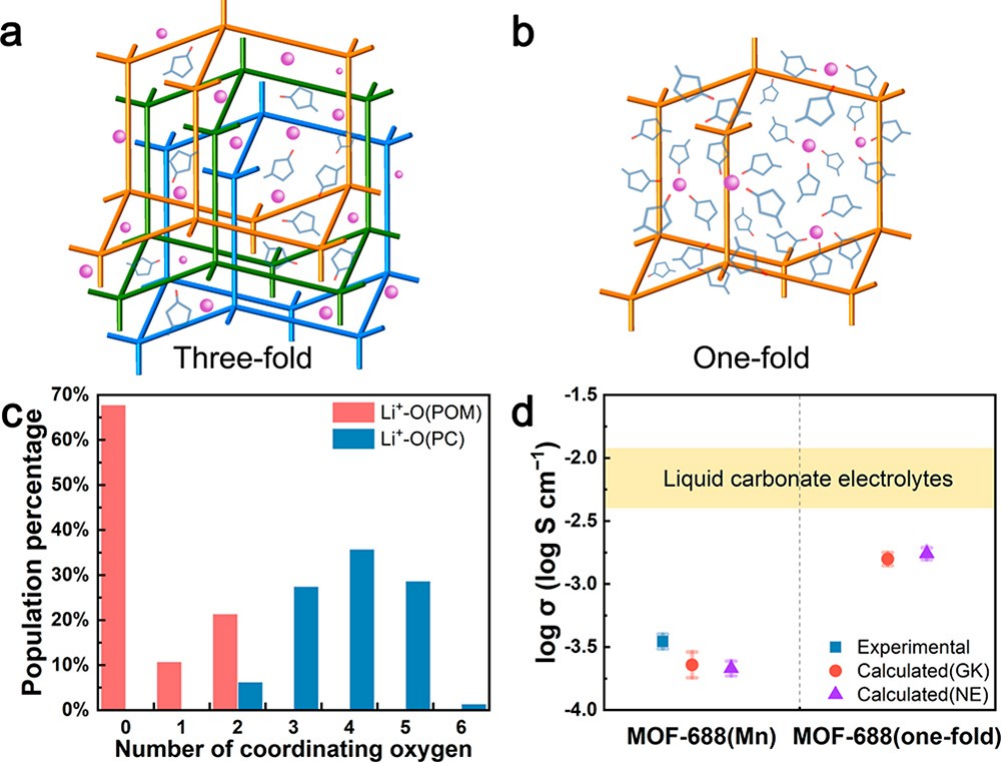
MOF-688(one-fold) design. Schemes of MOF-688 QSSEs with (a) three-fold and (b) proposed one-fold interpenetrating structures. (c) Coordination number of Li^+^-O(POM) and Li^+^-O(PC) in MOF-688(one-fold). (d) Comparison of ionic conductivities of MOF-688(Mn), MOF-688(one-fold), and the conductivity range of liquid carbonate electrolytes (highlighted).

To corroborate the hypothesized design, we modeled a noninterpenetrating MOF-688 denoted as MOF-688(one-fold) (Figure 3b). After the same insertion process, a POM/PC ratio of 8:355 was obtained (Figure S6). The apparent and effective Li^+^ concentrations are both 0.7 mol L^–1^, comparable to the usual concentration (1.0 mol L^–1^) of conventional liquid electrolytes. Further analysis of solvation structure reveals that more than 60% Li^+^ are fully solvated by PC (Figure 3c, Figure S7). The boost in solvent-separated Li^+^ is especially favorable for the uncorrelated Li^+^ diffusion in electrolytes.^6^ Using the same methods, the GK and NE conductivities of MOF-688(one-fold) are calculated to be 1.58 and 1.74 mS cm^–1^, respectively. The theoretical prediction (Figure 3d) is almost an order of magnitude higher than the conductivity of MOF-688(Mn) and other anionic MOF-based electrolyte,^9,10^ and significantly narrows the gap with typical liquid electrolyte conductivities (5–10 mS cm^–1^).^30,31^

Furthermore, specific contributions from the tethered and solvated Li^+^ to the total ionic conductivity were calculated using the NE equation and Li^+^ self-diffusion coefficients (Figure S11), in which the solvated Li^+^ contributed to 64% of the total ionic conductivity. Therefore, the ionic conduction in MOF-688(one-fold) can be mainly attributed to the solvated Li^+^ diffusion, providing a different ionic conduction mechanism with significantly improved conductivity. Moreover, unlike polymers which can become solvated and lose their mechanical strength after mixing with organic electrolyte,^32^ mechanical properties of MOFs can even be enhanced when solvent fills the pores.^33,34^ The bulk modulus (Voigt average) of MOF-688(one-fold) is calculated to be 1.1 and 2.4 GPa before and after adding PC, respectively. This is 1 to 2 orders of magnitude higher than that of poly(ethylene oxide) gel polymer electrolytes (a common QSSE)^35−37^ and retained about 1/3 of the 3-fold interpenetrating MOF-688(Mn) (SI Section 7).

To actually synthesize MOF-688(one-fold) with a much larger pore size than MOF-688(Mn), large and appropriately matching template guests or cations are required to support the reticulation of organic and inorganic building blocks. We envision that screening methods based on first-principles calculations^38^ and machine learning of suitable synthetic pathways^39,40^ may help identify template candidates.

## Supporting Information Available

Experimental and computational details including materials and synthetic procedures, single-crystal X-ray analysis, molecular dynamics and grand canonical Monte Carlo simulations, coordination analysis, conductivity calculation, elastic constants calculation, electrostatic potential surface analysis, and concentration analysis. (PDF)
