# Supplementary material for: Ionic Conduction Mechanism and Design of Metal–Organic Framework Based Quasi-Solid-State Electrolytes
Source: J Am Chem Soc. 2022 Jun 14;144(30):13446–50. doi: 10.1021/jacs.2c03710 (PMC9377385; doi:10.1021/jacs.2c03710)
Supplement: Supplementary file 1 — ja2c03710_si_001.pdf [file ja2c03710_si_001.pdf]

Supporting Information

Ionic conduction mechanism and design of metal–organic framework based quasi-solid-state electrolytes

Tingzheng Hou,[a,b,‡] Wentao Xu,[c,‡] Xiaokun Pei,[c] Lu Jiang,[a,d] Omar M. Yaghi,[c,*] and Kristin A. Persson[a,e,*]

[a]Department of Materials Science and Engineering, University of California Berkeley, 210 Hearst Mining Building, Berkeley, California 94720, United States

[b]Energy Technologies Area, Lawrence Berkeley National Laboratory, Berkeley, California 94720, United States

[c]Department of Chemistry, University of California, Berkeley, California 94720, United States

[d]Materials Sciences Division, Lawrence Berkeley National Laboratory, Berkeley, CA 94720, United States

[e]The Molecular Foundry, Lawrence Berkeley National Laboratory, Berkeley, California 94720, United States

‡T. Hou and W. Xu contributed equally.

Corresponding Author

* Kristin A. Persson: kapersson@lbl.gov

* Omar M. Yaghi: yaghi@berkeley.edu

Table of Contents

Section 1. Materials and Synthetic Procedures......3

Section 2. Single-Crystal X-ray Analysis......5

Section 3. Molecular dynamics and grand canonical Monte Carlo simulations......9

Section 4. Coordination Analysis......14

Section 5. Conductivity Calculation......15

Section 6. Partial contribution of tethered and freely solvated Li^+ to transport properties.19

Section 7. Elastic constants calculation......21

Section 8. Ionic conductivity as a function of temperature......24

Section 9. Electrostatic potential (ESP) surface of $AlMo_6$ cluster......25

Section 10. Concentration analysis......26

References......27

Section 1. Materials and Synthetic Procedures

Chemicals.

All starting materials and solvents, unless otherwise specified, were obtained from Sigma-Aldrich Chemicals, and used without further purification. $[N(C_4H_9)_4]_3[AlMo_6O_{18}(OH)_3\{(OCH_2)_3CNH_2\}]$ was synthesized according to literature reports.[1-2]

Analytical techniques and instruments.

Inductively Coupled Plasma-Optical Emission Spectrometry (ICP-OES) experiments were carried out on a Perkin Elmer Optima 7000 spectrometer. The samples were digested in a solution of 67–70% nitric acid (1.0 mL) and 48% hydrofluoric acid (0.1 mL) for 15 min and diluted with Milli-Q water to 35.00 mL before the measurement.

Electrochemical measurements were carried out using a BioLogic VMP3 potentiostat/galvanostat. The pellet of electrolyte was prepared in a split-able test cell (MTI Corporation) by hand pressing MOF-688 into a PTFE cylindrical die with 10 mm diameter in an Ar filled glovebox. The pellet was then sandwiched between two stainless steel blocking electrodes. The thickness of the pellet is 1.0 mm determined by the thickness of the mode.

Single crystal X-ray diffraction data was collected using synchrotron radiation on beamline 12.2.1 at the Advanced Light Source (ALS) at Lawrence Berkeley National Lab (LBNL). Beamline 12.2.1 is equipped with Bruker D8 and a PHOTON-II CMOS detector. The radiation is monochromated using silicon (111).

Synthetic Procedures.

$[N(C_4H_9)_4]_3[AlMo_6O_{18}\{(OCH_2)_3CNH_2\}_2]$: $[N(C_4H_9)_4]_3[AlMo_6O_{18}(OH)_3\{(OCH_2)_3CNH_2\}]$ (8.94 g) and tris(hydroxymethyl) aminomethane (0.61 g) was dissolved in 100 mL of ethanol and refluxed overnight. The reaction mixture was filtered to remove the precipitate. Ether was diffused into the filtrate and transparent crystals of $[N(C_4H_9)_4]_3[AlMo_6O_{18}\{(OCH_2)_3CNH_2\}_2]$ were obtained (yield 60% based on Mo). Elemental analysis: for C56H124AlMo6N5O24 Calcd. C 36.27%, H 6.74%, N 3.78%, Al 1.46%, Mo 31.05%, Found C 35.99%, H 6.73%, N 3.76%, Al 1.93%, Mo 34.40%.

MOF-688(Al): MOF-688(Al) was synthesized under similar condition of MOF-688(Mn).[3] 1000 mg of $[N(C_4H_9)_4]_3[AlMo_6O_{18}\{(OCH_2)_3CNH_2\}_2]$ ($AlMo_6$) and 110 mg of tetrakis(4-formylphenyl)methane (TFPM) were dissolved in a mixture of 10 mL anhydrous acetonitrile and 10 mL of anhydrous dioxane. Then 500 µL of acetic acid was added, and the solution was evenly distributed into four 20 mL vials. The vials were placed in an oven at 100 °C for 2 days. The resulting solid was collected *via* filtration. Solvent exchange was performed with anhydrous *N*,*N*-dimethylformamide at room temperature (40 mL × 3) over 24 hours, and with anhydrous acetonitrile at room temperature (40 mL × 6) over 2 days. Elemental analysis: for C141H260Al2Mo12N10O48 Calcd. C 41.62%, H 6.44%, N 3.44%, Al 1.33%, Mo 28.30%, Found C 39.82%, H 6.56%, N 3.50%, Al 1.76 %, Mo 30.04 %.

Ion exchange on MOF-688(Al): Ion exchange on MOF-688(Al) was carried out using the same procedure reported previously.[3] 200 mg of MOF-688(Al) was immersed in 5 mL of 1 M acetonitrile solution of LiTFSI at room temperature. The solution was changed three times per day over two days. Then the ion exchanged MOF-688 was immersed in fresh anhydrous acetonitrile

(40 mL × 6) over two days to remove excess lithium salt in the pores. ICP-OES was carried out with the ion exchanged MOF-688 and a 3:1 ratio was confirmed between Li and $AlMo_6$.

Section 2. Single-Crystal X-ray Analysis

A crystal of MOF-688(Al) was mounted on MiTeGen® kapton loops and placed in a 100 K nitrogen cold stream from Oxford Cryosystem 800. The raw data were processed with the Bruker APEX3 software package.[4] The data were first integrated using the SAINT V8.38A and then corrected for absorption with SADABS 2016/2 routines.[5] The structures were solved by intrinsic phasing (SHELXT-2018/02) and the refinement was done by full-matrix least squares on F^2 (SHELXL-2018/03), using the OLEX2 software package.[6-8]

The diffraction images showed significant nebula-like diffuse scattering signals, which influenced the integration of Bragg signals and thus the accuracy of Bragg intensities. We noticed that the integration of the weak Bragg peaks was strongly influenced by the diffuse signals, evident by that the completeness can vary from ~ 80% to the reported value (91.5%) by changing background determination methods. We speculate the weak Bragg signals were partly surpassed by diffuse signals in the background determination method for the reported dataset as well. The automatic space group determination by XPREP suggested $I4_1$/amd as the correct space group, however, the inspection into the preliminary structure solution in $I4_1$/amd showed that this symmetry is higher than the possible symmetries of the chemical units. Considering the major scatterer in the structure is the $AlMo_6$ cluster and the cluster is more ordered than TPM, $I4_1$/amd is likely to be a higher pseudo-symmetry that only reflects the symmetry of $AlMo_6$ clusters in crystals. Therefore, the actual space group was determined to be a lower space group, $I4_1$/a. The resolution of the dataset was determined by a combined criteria of R_{merge} and completeness. The completeness could be higher by cutoff to lower resolutions, but the low resolution put more severe effect in quality of

constructed electron densities. After comparing the refined datasets with different resolution cutoff, a cutoff at 1.08 angstrom was chosen with the R_{merge} at highest resolution shell < 40 %.

The anisotropic atom refinement on $AlMo_6$ cluster and the central carbon of TPM suggested that there is a collective disorder throughout the framework. The electron densities around the phenyl rings of TPM and the imine bonds were relatively smeared, suggesting the conformational disorder of both as two additional sources of disorder. The phenyl rings and the imine bonds were refined as two disordered positions, although in reality the number of disordered positions might be much more than two. Due to the strong disorder, anisotropic refinement on these disordered sites failed, and thus the atoms from phenyl rings and imines were refined isotropically. SADI was applied to align the imine bond distances from two disordered sites. EADP was used to constrain the displacement parameters of imine carbons of two disordered positions. DFIX was applied to constrain one of the C_{imine}-C_{Ar} bond distance that was disturbed by the low definition of the electron densities around. Solvent mask was applied at the end of refinement.

Table S1. Crystal data for MOF-688(Al).

Empirical formula	$C_{11.25}H_5Al_{0.5}Mo_3NO_{12}$
Formula weight	647.47
Temperature/K	100
Space group	$I4_1/a$
a/Å	37.966(4)
b/Å	37.966(4)
c/Å	13.5382(16)
α/°	90
β/°	90
γ/°	90
Volume/$Å^3$	19514(4)
Z	16
ρ_{calc} (g/cm^3)	0.882
μ/mm^{-1}	0.850
F(000)	4928.0
Crystal size/mm^3	0.04 × 0.04 × 0.015
Radiation	Synchrotron (λ = 0.7288 Å)
2Θ range for data collection/°	6.308 to 39.406
Index ranges	-35 ≤ h ≤ 35<br>-34 ≤ k ≤ 35<br>-11 ≤ l ≤ 11
Reflections collected	18405
Independent reflections	3715 [R_{int} = 0.0858, R_{sigma} = 0.0689]
Data/restraints/parameters	3715/4/220
Goodness-of-fit on F^2	1.117
Final R indexes [I>=2σ (I)]	R_1 = 0.0732, wR_2 = 0.1861
Final R indexes [all data]	R_1 = 0.0840, wR_2 = 0.1944
Largest diff. peak/hole / e $Å^{-3}$	0.35/-0.42

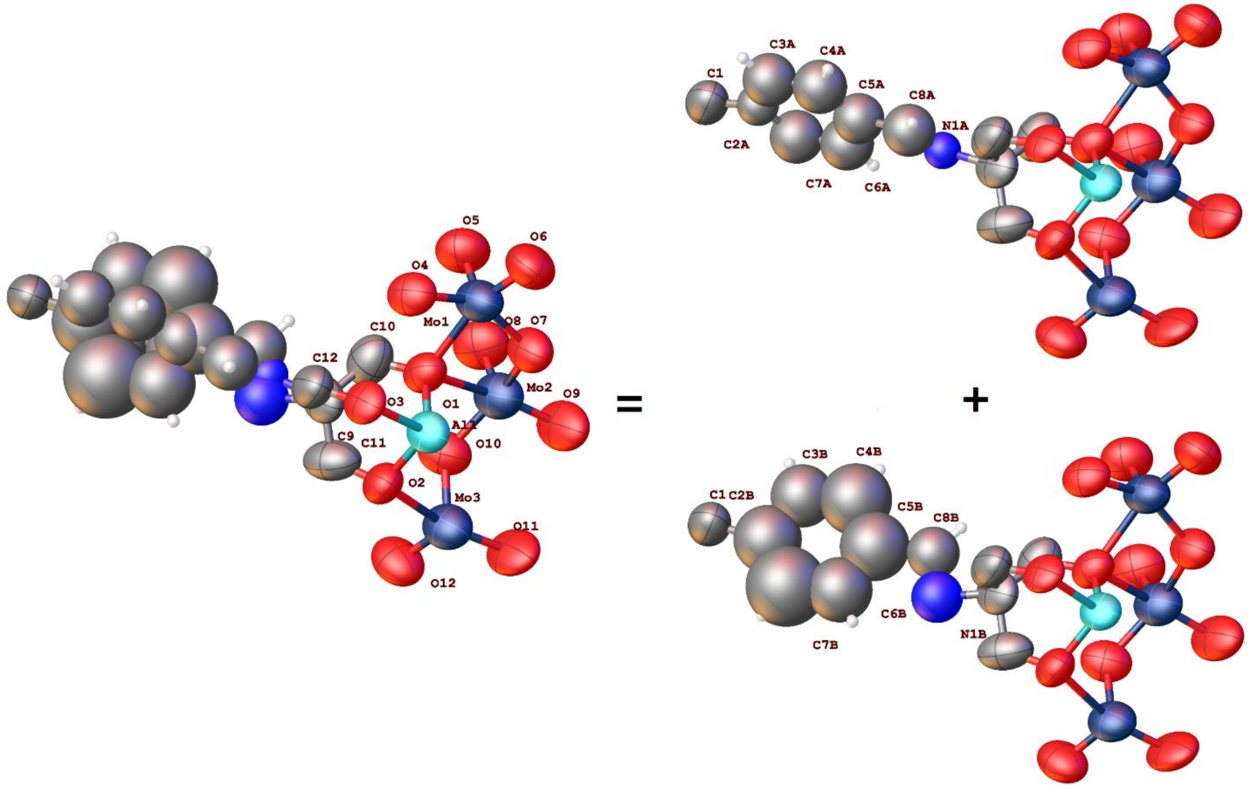

Figure S1. The asymmetric unit of MOF-688(Al) (left), and the separate illustration for two disordered positions (right). Thermal ellipsoids are plotted with 50% probability.

Section 3. Molecular dynamics and grand canonical Monte Carlo simulations

Structures

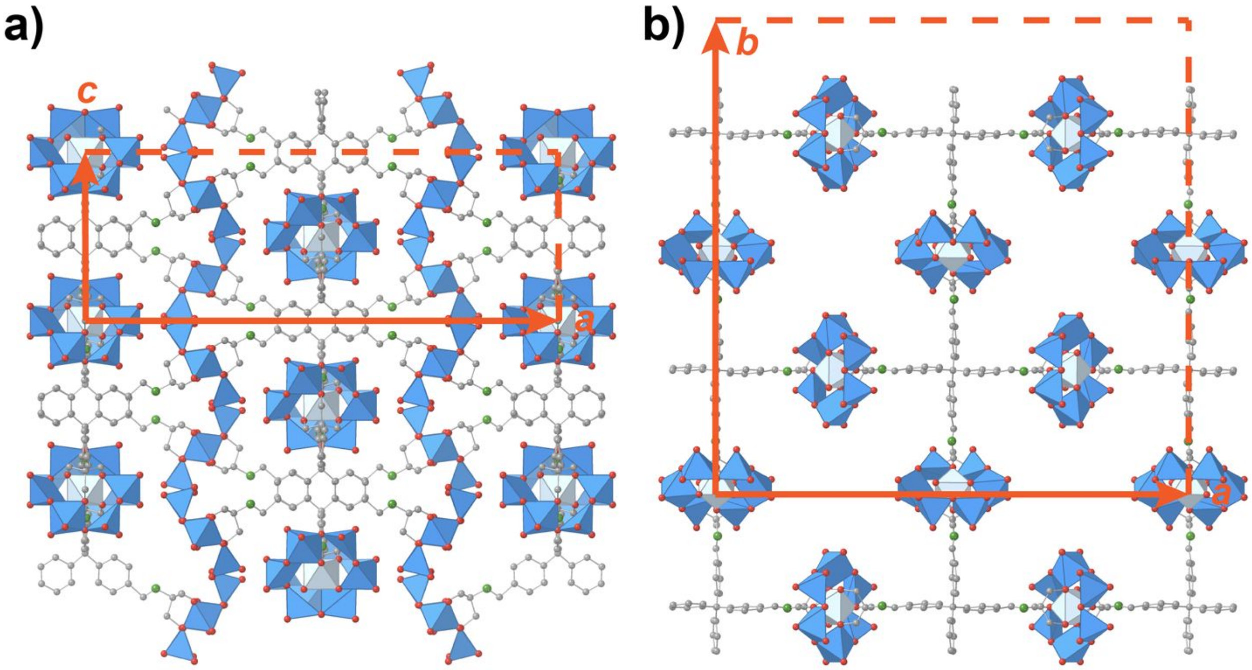

Figure S2. (a) The view of MOF-688(Mn/Al) along crystallographic *b* axis. (b) The view of MOF-688(Mn/Al) along crystallographic *c* axis. Color code: Mo, sky blue octahedra; central metal ion in POM (Mn/Al), light blue octahedra; oxygen, red; nitrogen, green; carbon, gray. Hydrogens are omitted for clarity.

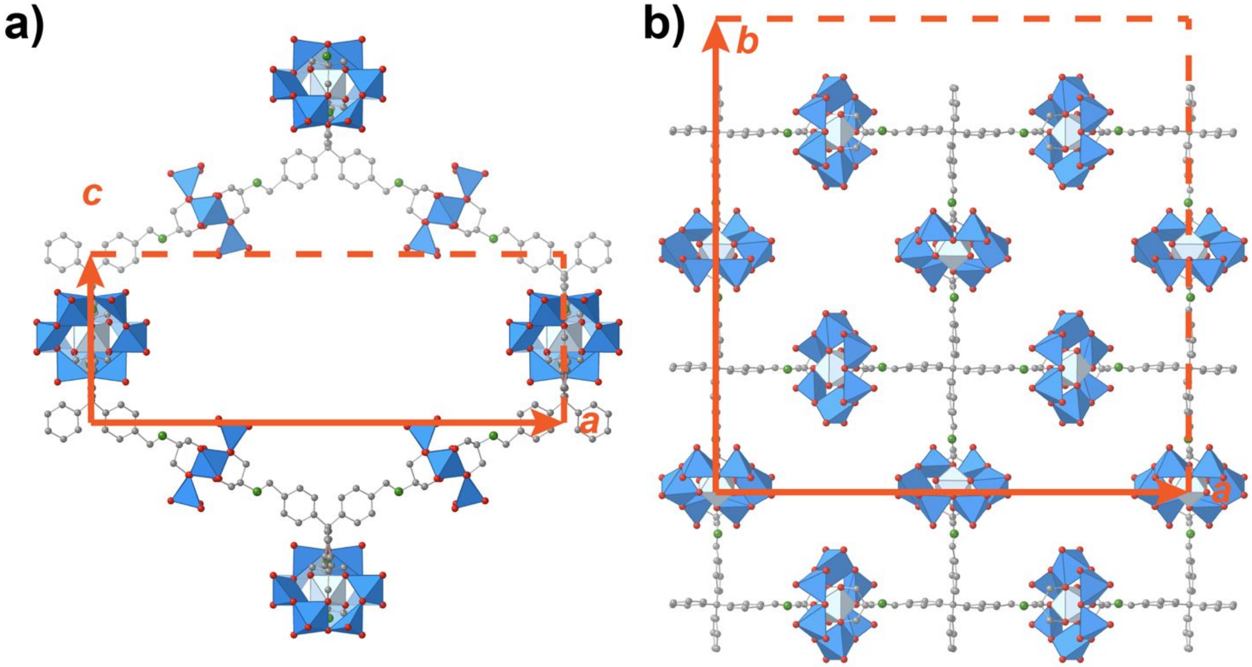

Figure S3. (a) The view of MOF-688(one-fold) along crystallographic *b* axis. (b) The view of MOF-688(one-fold) along crystallographic *c* axis. Color code: Mo, sky blue octahedra; central metal ion in POM (Mn), light blue octahedra; oxygen, red; nitrogen, green; carbon, gray. Hydrogens are omitted for clarity.

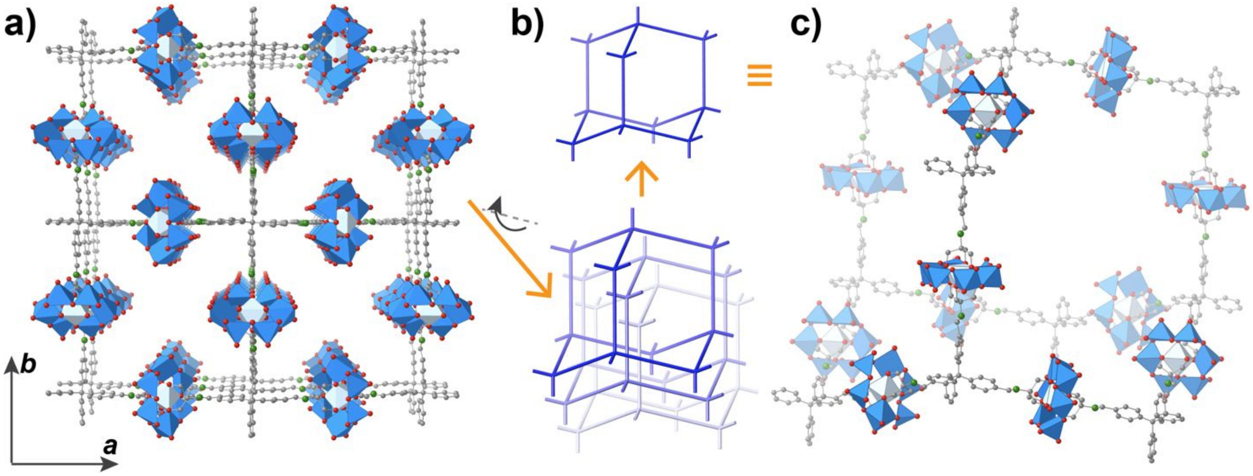

Figure S4. The (b) interpenetration relationship between (a) a MOF-688(Mn) and (c) a MOF-688(one-fold).

Simulation box The atomic position obtained from single crystal X-ray diffraction (SXRD) of MOF-688 was used as the initial structure to build a supercell containing 12 polyoxometalate (POM) clusters, denoted as MOF-688(Mn). In parallel, an isoreticular structure, termed MOF-688(Al), was modeled by substitute Mn^{3+} with Al^{3+}.

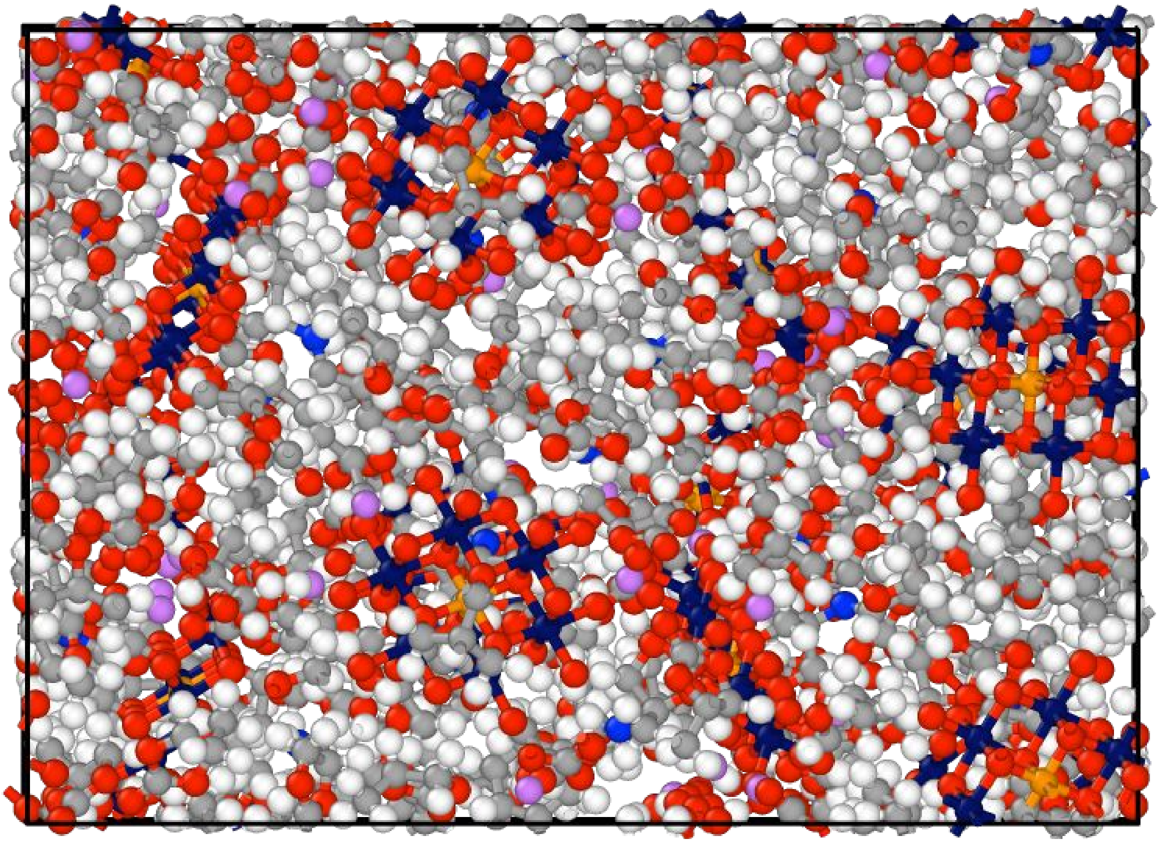

Figure S5. The simulation box of MOF-688(Mn). The carbon, hydrogen, oxygen, nitrogen, molybdenum, manganese, and lithium elements are represented by grey, white, red, blue, dark blue, orange, and purple, respectively.

Parameterization The nonpolarizable UFF[9] was utilized to parameterize the bonded and van der Waals interactions, which was generated using LAMMPS interface.[10] The bonded and non-bonded parameters for PC were obtained from the OPLS-AA force fields (Optimized Potentials for Liquid Simulations All Atom),[11-12] while those for Li^+ are taken from Jensen et al.[13]

Partial charges of the intrinsically anionic frameworks were fitted from first principles using the RESP method.[14-15] with B3LYP/6-31+G(d,p)//B3LYP/aug-cc-pvdz[16-18] level of theory for the organic linker and oxygen, manganese in the POM cluster, and B3LYP/LANL2TZ(f)//B3LYP/LANL2TZ(f)[19] level of theory for molybdenum in the POM cluster, respectively. Long-range electrostatic interactions were handled by the particle-particle particle-mesh (PPPM) solver with a grid spacing of 0.1 nm. A cutoff distance of 1.25 nm was used for electrostatic and 12–6 Lennard-Jones interactions. The fitted partial charges and the unit charge of Li^+ (+1) were then scaled by a factor of ε=0.6 to account for the fact that solvent–ion and ion–ion interactions are typically overestimated in nonpolarizable force fields[20-22].

Table S2. The fitted RESP atomic charges of the intrinsically anionic frameworks of MOF-688(Mn) and MOF-688(Al) after scaling by a factor of ε=0.6.

Atom types

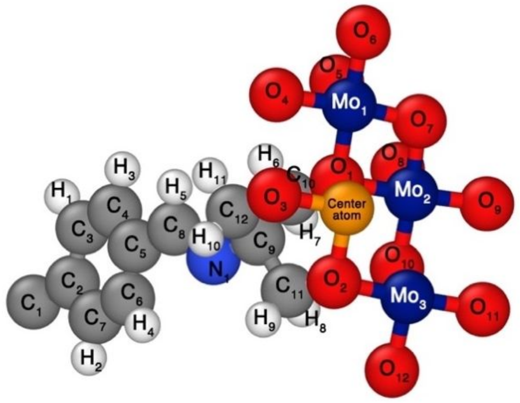

Atom type	MOF-688(Mn)	MOF-688(Al)
Li	0.6	0.6
C_1	-0.6444	-0.6504
C_2	0.2778	0.2778

C_3, C_7	-0.1368	-0.1368
H_1, H_2	0.0888	0.0888
C_4, C_6	-0.0876	-0.0876
H_3, H_4	0.0762	0.0762
C_5	0.0240	0.0240
C_8	0.135	0.1356
H_5	0.0078	0.0072
N_1	-0.4002	-0.4044
C_9	0.3132	0.3066
C_{10}, C_{11}, C_{12}	-0.1026	-0.1086
H_6, H_7, H_8, H_9, H_{10}, H_{11}	0.0468	0.0480
O_1, O_2, O_3	-0.0864	-0.0810
O_4, O_7, O_{10}	-0.4332	-0.4434
O_5, O_6, O_8, O_9, O_{11}, O_{12}	-0.3780	-0.3816
Mo_1, Mo_2, Mo_3	0.9564	0.9762
Center atom	0.0138 (Mn_1)	0.0132 (Al_1)

PC insertion Hybrid Molecular dynamics (MD) and grand canonical Monte Carlo (GCMC) simulations[23] were performed to insert the PC solvent into the pores of the MOF by exchanging molecules with an imaginary reservoir of PC. As shown in Figure S6, after at least 200 ns equilibration, the POM:PC ratio converged to 16:170 for MOF-688(Mn), and after at least 100 ns equilibration the POM:PC ratio converged to 8:355 for MOF-688(one-fold).

Simulation procedures The obtained PC-infused structure was then equilibrated for 2 ns in the isothermal-isobaric ensemble (T = 298 K, P = 1 bar) using the Parrinello–Rahman barostat followed with an annealing process. Subsequently, 50 ns production runs within the canonical ensemble (NVT) under a Nosé–Hoover thermostat were performed to obtain the Li^+ transport properties. Independent duplicate production runs were undertaken for the MOF-688(Mn), MOF-688(Al), and MOF-688(one-fold). Statistical uncertainties (error bars) were estimated by calculating the standard deviation of the independent 50 ns duplicates.

All molecular simulations were carried out with the LAMMPS (Large Scale Atomic/Molecular Massively Parallel Simulator) software package,[24] and quantum chemical calculations were performed using Gaussian 16.[25] Analysis of the ionic conductivity, coordination, density plot were undertaken using the Python package MDAnalysis[26] and mdgo,[27] and visualizations of the density plot were generated using the software Mathematica.[28]

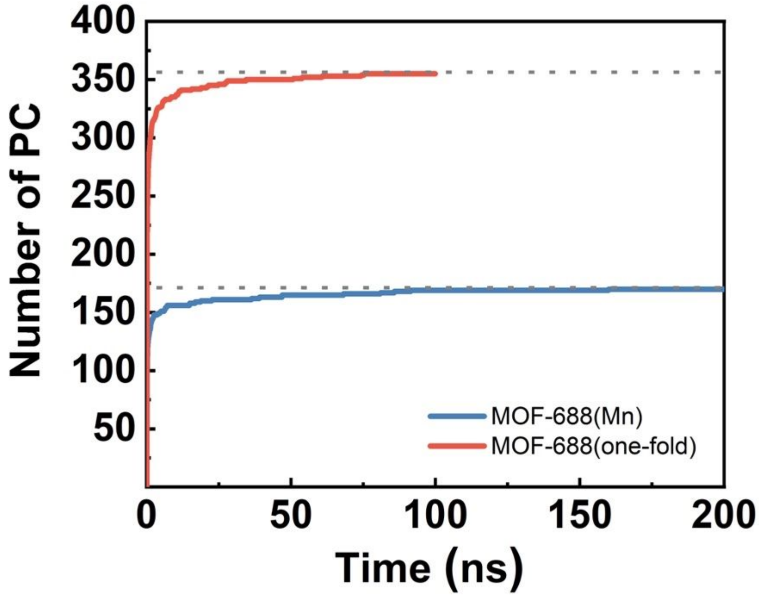

Figure S6. Insertion of the PC solvent during the hybrid Molecular dynamics (MD) and grand canonical Monte Carlo (GCMC) simulation. The number of PC converged to 170 after at least 200 ns of simulation time for MOF-688(Mn) and 355 after at least 100 ns of simulation time for MOF-688(one-fold).

Section 4. Coordination Analysis

The coordination states of (a) Li-O(POM), and (b) Li-O(PC) in MOF-688(Al), MOF-688(Mn), and MOF-688(one-fold) were identified from the Li-O radial distribution functions during the production runs (Figure S7). A lithium ion is considered coordinate to an oxygen of POM and PC if the oxygen is within the cutoff distance defined by the minimum after the first peak in the Li-O(POM) RDF (3.2 Å for all models). The distribution of coordination number (main text, Figure 1a, b) can be then calculated accordingly, which is averaged over all hopping events in each simulation.

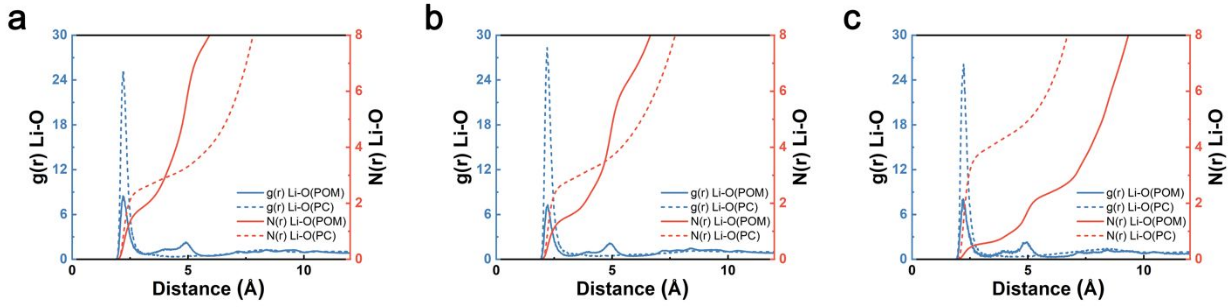

Figure S7. Li-O radial distribution functions g(r) (left axis) and coordination numbers N(r) (right axis) of MOF-688(Al), MOF-688(Mn), and MOF-688(one-fold).

The Li^+ density plot on the $MnMo_6$ surface was obtained by counting 100,000 randomly sampled Li^+ coordinates during the MD simulation and averaged over all 16 POM clusters. Li^+ coordinates were linearly transformed from the Cartesian coordinate system to a Mn-centered spherical coordinate system according to the symmetry of the POM cluster before taking the average. The plot uses an 8 Å × 8 Å ×5 Å mesh with each mesh division 0.2 Å equally spaced.

We implemented a one-sided independent two-sample t-test to validate that the observed changes of coordination number during hopping are not due to fluctuation.

For two different time steps in each trajectory shown in main text Figure 1e,f , given that the sample sizes remain identical, and assuming that the distributions of coordination number at different time steps have the same variance, the t statistic to test whether the means of coordination number at the two time steps are different can be calculated as follows:

$$t = \frac{\bar{X}_1 - \bar{X}_2}{\sqrt{\frac{s_{X_1}^2 - s_{X_2}^2}{n}}}, \ (R1)$$

where $\bar{X}_1$ and $\bar{X}_2$ are the mean coordination number at the two time steps, $n = n_1 = n_2$ are the trajectory sample sizes, and $s_{X_1}^2$ and $s_{X_2}^2$ are the unbiased estimators of the variances of the coordination number the two time steps.

Two sets of samples of coordination number of Li^+-O(POM) and Li^+-O(PC) in MOF-688(Mn) and MOF-688(Al) at −1000 ps (as a representative of the bulk average) and at around 0 ps (the spike during hopping) in main text Figure 1e,f are chosen to perform the t testing. The statistics and calculated results are listed in Table S3.

Table S3. The means ($\bar{X}$), standard deviations (s), trajectory sample size (n), degree of freedom ($2n-2$), t statistic, and the corresponding one-sided p-value of coordination number Li^+-O(POM) and Li^+-O(PC) in MOF-688(Mn) and MOF-688(Al) at −1000 ps and at the time step of hopping.

Material	Coordination number sample	$\bar{X}$	s	n	$2n-2$	t	p-value
MOF-688 (Mn)	Li^+-O(PC) (at −1000 ps)	3.030	0.8111	48	94	2.147	0.0172
	Li^+-O(PC) (at 170 ps)	3.401	0.8844				
	Li^+-O(POM) (at −1000 ps)	1.3080	0.8910	48	94	3.811	0.000123
	Li^+-O(POM) (at 40 ps)	0.6549	0.7844				
MOF-688 (Al)	Li^+-O(PC) (at −1000 ps)	2.9605	0.8029	77	152	5.373	7.75×10^{-8}
	Li^+-O(PC) (at 50 ps)	3.4912	0.9155				
	Li^+-O(POM) (at −1000 ps)	1.2500	0.7972	77	152	7.400	6.83×10^{-13}
	Li^+-O(POM) (at 40 ps)	0.6114	0.7048				

As shown in the table, for all the four trajectories in Figure 1e,f, the p-value obtained from the t testing is statistically significant ($p < 0.05$). Therefore, we conclude that the mean coordination number during hopping is significantly different from the bulk average.

Section 5. Conductivity Calculation

Firstly, the GK conductivity was rigorously computed using the following Green–Kubo (GK) relations

$$\sigma_{GK} = \frac{1}{6k_BTV}\lim_{t\to\infty}\frac{d}{dt}\left\langle\sum_{i=1}^{N}\sum_{j=1}^{N}q_iq_j[\mathbf{r}_i(t)-\mathbf{r}_i(0)]\cdot[\mathbf{r}_j(t)-\mathbf{r}_j(0)]\right\rangle, \quad \text{(S1)}$$

where k_B is the Boltzmann constant, T is temperature, V is the cell volume, q_i is the charge of species i, $\mathbf{r}_i(t)$ is the coordinates of species *i* at time *t*.

The NE conductivity can be obtained from the self-diffusion coefficient using the Nernst–Einstein (NE) equation from the diffusivity of Li^+

$$\sigma_{NE} = \frac{F^2}{RT}(c_+{q_+}^2D_+ + c_-{q_-}^2D_-) = \frac{F^2}{RT}c_+D_+, \quad \text{(S2)}$$

where σ_{NE} is the ionic conductivity, c_+ and c_- are the bulk molar concentrations of the cation and anion, q_+ and q_- are the charge of the cation and anion, and D_+ and D_- are the self-diffusion coefficients of the cation and anion. D_- is assumed to be 0. D_+ for Li^+ can be obtained by calculating the slope of the mean square displacement (MSD, $\mathbf{r}^2(t)$) over time using the Stokes–Einstein relation:[29]

$$D_+ = \frac{1}{6}\lim_{t\to\infty}\frac{\mathrm{d}}{\mathrm{d}t}\langle\mathbf{r}^2(t)\rangle, \quad \text{(S3)}$$

where $\mathbf{r}$ is the position vector of Li^+ at time t. The NE equation has been widely used in describing the correlation of diffusivity and conductivity in porous systems and showed excellent agreement with experimental results.[30-31] The NE equation assumes the solution behaves ideally with no correlations between species, and the ionic conductivity can be calculated using only the self-diffusion coefficients of charged species. In addition, if the sum of all the distinct terms of the

ionic conductivity is zero (see below), the resulting conductivity also follows Nernst−Einstein or ideal solution behavior, which is the case for our MOF-based QSSEs.

The resulting coefficient in the angular brackets in Eq. S3 is fitted from a least-squares minimization for a straight line for a time period in the diffusion regime and then averaged over at least three independent realizations of the same system. Single particle dynamics obtained from MSD can be divided into three regimes (1) ballistic at short timescales when the ions have not interacted much with their neighbors (MSD $\propto t^2$) (2) subdiffusive at intermediate times where ions rattle inside a cage formed by their neighbors (MSD $\propto t^\alpha$, $0 < \alpha < 1$), and when the ions escape from these cages, they reach the diffusive or Fickian regime at increased timescales (MSD $\propto t$). In this work we only captured the behavior in the diffusive regime. As is the case with diffusion coefficient calculations, a mathematically rigorous analysis of the GK conductivity requires the effective "mean square displacement" enclosed in the angular brackets of Eq. S1 to be linear in time. The simulations performed here all reached the linear regime as with the diffusion coefficient analysis above. Log-log plots of MSD and the effective "mean square displacement" in Eq. S1 from representative simulations of MOF-688(Mn), MOF-688(Al) and MOF-688(one-fold) are shown in Figure S8 and S9 to demonstrate this linear behavior for both self-diffusion coefficients and ionic conductivities calculation.

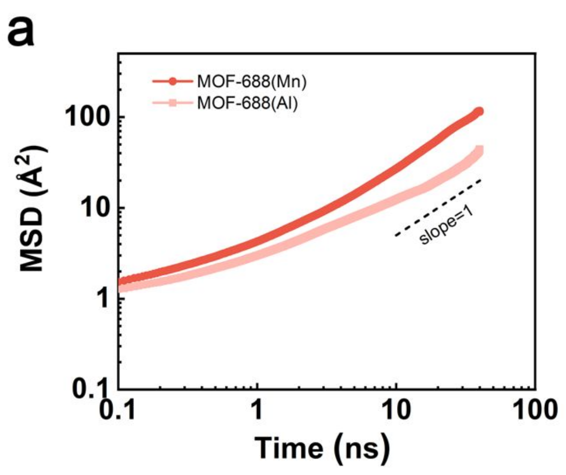

Figure S8. Representative examples of the linear diffusion behavior of MOF-688(Mn) and MOF-688(Al) required to calculate (a) self-diffusion coefficients, and (b) ionic conductivity. A slope of one (corresponding to linear data on a log-log plot) is indicated on each plot. The effective "mean square displacement" on the y-axis of panel (b) is the quantity in angular brackets in Eq. S1.

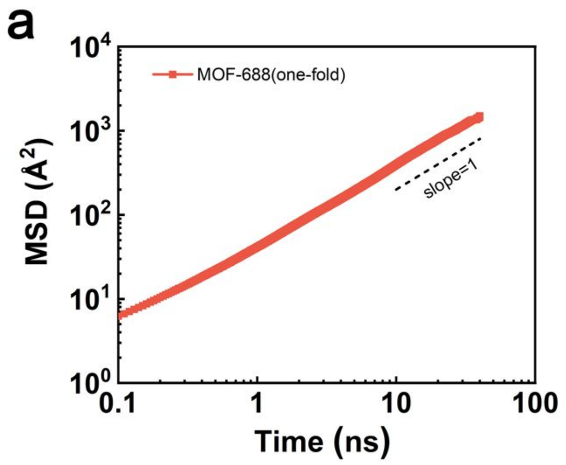

Figure S9. Representative examples of the linear diffusion behavior of MOF-688(one-fold) required to calculate (a) self-diffusion coefficients, and (b) ionic conductivity. A slope of one (corresponding to linear data on a log-log plot) is indicated on each plot. The effective "mean square displacement" on the y-axis of panel (b) is the quantity in angular brackets in Eq. S1.

The simple hopping model is based on the precondition of the NE equation and a further assumption that the self-diffusion coefficients of charged species are mainly contributed by the intermittent hopping. The hopping diffusion coefficient D under the theoretical framework of random walk can be expressed as

$$D = \frac{\Gamma a^2}{b}, \quad \text{(S4)}$$

where Γ is the hopping frequency of successful jumps, a is the hopping distance between two neighboring binding sites of Li^+, and b is a geometry factor of 2, 4, or 6 for one-, two-, or three-dimensional diffusion, respectively. Li^+ within a certain distance of a POM cluster is considered tethered to that POM cluster. When analyzing the hopping events, the hopping cutoff distance is chosen as half of the averaged distance between the centers neighboring POM clusters. Using the cutoff distance as a criterion, a successful hop is defined as one Li^+ moves beyond the hopping cutoff distance of a previously tethered POM cluster and binds to another POM cluster. The hopping distance was obtained by averaging the distance between neighboring binding sites in hopping events. Thus, the diffusion coefficient can be estimated based on the hopping frequency and hopping distance. Incorporating the hopping diffusion coefficient into Eq. S2, we obtain the hopping conductivity.

The Onsager transport equations[32] has been demonstrated to rigorously analyze and quantify the correlations of ion motion in gel polymer electrolytes (GPEs, another type of QSSEs). Considering the structural similarity of MOF-based electrolyte and GPEs, we utilized the Onsager framework to quantitatively determine the degree of correlated ion motions in the material.

According to the Onsager transport theory, the ionic conductivity can be decomposed into separate terms, each corresponding to a different type of uncorrelated or correlated ion motion, namely the

cation-self (σ^{s}_{cat}), anion-self (σ^{s}_{an}), cation-distinct (σ^{d}_{cat}), anion-distinct(σ^{d}_{an}), and cation-anion-distinct ($\sigma^{d}_{cat,an}$) conductivities.[33]

$$\sigma = \sigma^{s}_{cat} + \sigma^{s}_{\mathrm{an}} + \sigma^{\mathrm{d}}_{cat} + \sigma^{d}_{\mathrm{an}} + \sigma^{d}_{cat,an}. \quad \text{(R2)}$$

These various conductivity contributions are defined as:

$$\sigma^{s}_{cat} = \frac{1}{6k_BTV}\lim_{t\to\infty}\frac{d}{dt}\left\langle\sum_{i=1}^{N}\sum_{j=1}^{N} q^{2}_{i_{cat}}\left[\mathbf{r}_{i_{cat}}(t) - \mathbf{r}_{i_{cat}}(0)\right]\cdot\left[\mathbf{r}_{i_{cat}}(t) - \mathbf{r}_{i_{cat}}(0)\right]\right\rangle, \quad \text{(R3)}$$

$$\sigma^{s}_{an} = \frac{1}{6k_BTV}\lim_{t\to\infty}\frac{d}{dt}\left\langle\sum_{i=1}^{N}\sum_{j=1}^{N} q^{2}_{i_{an}}\left[\mathbf{r}_{i}(t) - \mathbf{r}_{i}(0)\right]\cdot\left[\mathbf{r}_{j}(t) - \mathbf{r}_{j}(0)\right]\right\rangle, \quad \text{(R4)}$$

$$\sigma^{d}_{cat} = \frac{1}{6k_BTV}\lim_{t\to\infty}\frac{d}{dt}\left\langle\sum_{i=1}^{N}\sum_{j=1}^{N} q_{i_{cat}}q_{j_{cat}}\left[\mathbf{r}_{i}(t) - \mathbf{r}_{i}(0)\right]\cdot\left[\mathbf{r}_{j}(t) - \mathbf{r}_{j}(0)\right]\right\rangle, \quad \text{(R5)}$$

$$\sigma^{d}_{an} = \frac{1}{6k_BTV}\lim_{t\to\infty}\frac{d}{dt}\left\langle\sum_{i=1}^{N}\sum_{j=1}^{N} q_{i_{an}}q_{j_{an}}\left[\mathbf{r}_{i}(t) - \mathbf{r}_{i}(0)\right]\cdot\left[\mathbf{r}_{j}(t) - \mathbf{r}_{j}(0)\right]\right\rangle, \quad \text{(R6)}$$

$$\sigma^{d}_{cat,an} = \frac{1}{6k_BTV}\lim_{t\to\infty}\frac{d}{dt}\left\langle\sum_{i=1}^{N}\sum_{j=1}^{N} q_{i_{cat}}q_{j_{an}}\left[\mathbf{r}_{i}(t) - \mathbf{r}_{i}(0)\right]\cdot\left[\mathbf{r}_{j}(t) - \mathbf{r}_{j}(0)\right]\right\rangle, \quad \text{(R7)}$$

where k_B is the Boltzmann constant, T is temperature, V is the cell volume, $q_{i_{cat}}$, $q_{j_{cat}}$, $q_{i_{an}}$, $q_{j_{an}}$ are the charges of cation or anion *i, j*, $\mathbf{r}_i(t)$ is the coordinates of species *i* at time *t*. The two self-conductivity terms (σ^{s}_{cat} and σ^{s}_{an}) yield the conductivity from completely uncorrelated ion motion. The distinct terms (σ^{d}_{cat}, σ^{d}_{an}, $\sigma^{d}_{cat,an}$) capture the ion−ion correlations between pairs of cations, pairs of anions, and cation−anion pairs, respectively. If the sum of all the distinct terms is zero, then the resulting conductivity follows Nernst−Einstein or ideal solution behavior. Here, we calculated the corresponding contributions of a 50 ns MD trajectory of MOF-688(Mn).

Table S4. Contributions of each type of uncorrelated (self) or correlated (distinct) ion motion to the total ionic conductivity of MOF-688(Mn).

Type	Conductivity (mS cm^{-1})
σ_{cat}^{s}	0.173
σ_{an}^{s}	0.009
σ_{cat}^{d}	0.216
σ_{an}^{d}	0.223
$\sigma_{cat,an}^{d}$	-0.218
σ	0.185

As shown in Table S4, the total ionic conductivity ($\sigma =$ 0.185 mS cm^{-1}) is mostly contributed by the uncorrelated cation motion ($\sigma_{cat}^{s} =$ 0.173 mS cm^{-1}). Notably, even though the correlated cation motion term is even larger ($\sigma_{cat}^{d} =$ 0.216 mS cm^{-1}), it almost cancels with the cation−anion correlation term ($\sigma_{cat,an}^{d} =$ −0.218 mS cm^{-1}). This is due to the presence of a large portion (about 93%) of Li^+ that is tethered to the anionic framework. The bounded Li^+ ions and framework are constrained to move together for the lifetime of the coordination. Thus, the correlated motion cation is mainly due to the constrained movement of Li^+ ions that are tethered to the framework, which does not contribute to the total ionic conductivity. Therefore, we conclude that the ionic conductivity is mainly attributed to the uncorrelated cation jump, and the correlated cation motion is insignificant.

In addition, the Li^+ transference number can be further calculated using the following equation:[34]

$$t_{Li^+} = \frac{\sigma_{cat}^{s} + \sigma_{cat}^{d} + \sigma_{cat,an}^{d}}{\sigma_{cat}^{s} + \sigma_{cat}^{d} + \sigma_{an}^{s} + \sigma_{an}^{d} + 2\sigma_{cat,an}^{d}}$$

According to the above results, t_{Li^+} of the 50 ns MD trajectory of MOF-688(Mn) is calculated to be 0.92, which agree well with the experimentally measured value 0.87.

Section 6. Partial contribution of tethered and freely solvated Li^+ to transport properties

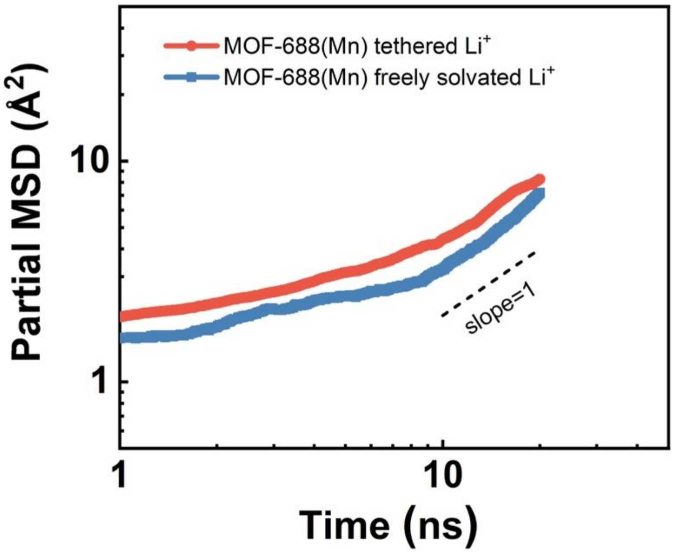

Figure S10. Self-diffusion coefficients of tethered and freely solvated Li^+ in MOF-688(Mn).

Similar to the coordination analysis, we have differentiated Li^+ as tethered, if it is within a cutoff distance of the POM clusters, or otherwise solvated. The cutoff distance is chosen as 3.2 Å for all models, which is the minimum after the first peak in the Li-O(POM) RDF.

When calculating the self-diffusion coefficients of tethered and solvated Li^+, we first determine the state (tethered or solvated) of Li^+ according to the coordinates at each individual time step (every 10 ps). Then, we cut each Li^+ trajectory into slices where Li^+ remains the same state within the slice. The slices with only one time step are excluded. Finally, using all the trajectory slices of tethered Li^+ and solvated Li^+, we calculate the self-diffusion coefficients of tethered Li^+ and solvated Li^+, respectively, by averaging over all trajectory slices of the same state, over all Li^+, over all time origins, and over all parallel runs.

The mean square displacement (MSD) of Li^+ in MOF-688(Mn) was calculated according to its coordination states (Figure S10). The self-diffusion coefficients of the tethered (Li^+ coordinated to POMs) and freely solvated Li^+ (Li^+ not coordinated to POMs) were then fitted from the MSD plot. Therefore, the specific contributions from to the total ionic conductivity can be obtained using the NE equation and the self-diffusion coefficients. For MOF-688(Mn), the calculated self-diffusion coefficients of the tethered and freely solvated Li^+ were 8.6 and 8.3 10^{-13} m^2 s, respectively. This indicates that the diffusion of freely solvated Li^+ is not the primary conduction mechanism.

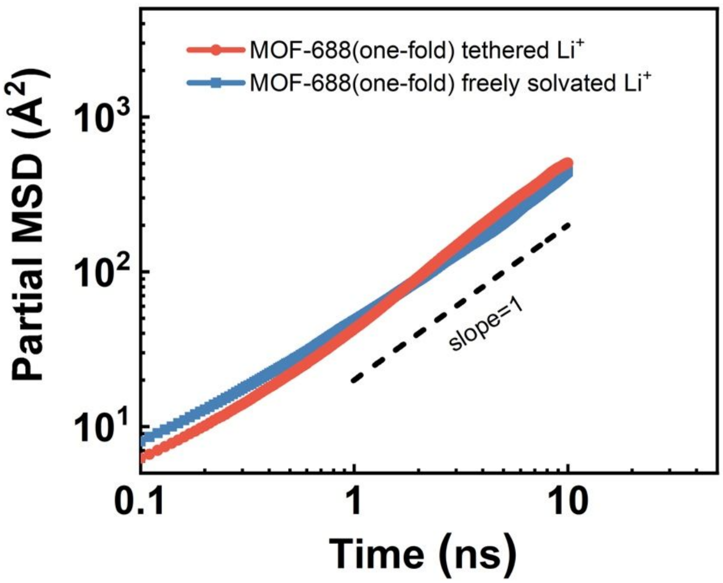

Figure S11. Self-diffusion coefficients of tethered and freely solvated Li^+ in MOF-688(one-fold).

The specific contributions from tethered and freely solvated Li^+ to the total ionic conductivity were calculated to be 0.71 and 1.24 mS cm^{-1}, respectively (Figure S11). Despite the minor discrepancy due to different statistical treatments, the summation of the two terms (1.95 mS cm^{-1}) agrees well with the NE conductivity calculated from the total self-diffusion coefficients of Li^+ (1.74 mS cm^{-1}). Note that the different treatment between the two conductivity calculation regimes accounts for

this minor discrepancy. The displacement of Li^+ at hopping steps when Li^+ changes state between the two is excluded when calculating the specific ionic conductivity contributions.

Section 7. Elastic constants calculation

In Voigt notation, the general stress-strain relationship is shown in Eq. S5:

$$\begin{pmatrix} C_{11} & C_{12} & C_{13} & C_{14} & C_{15} & C_{16} \\ C_{12} & C_{22} & C_{23} & C_{24} & C_{25} & C_{26} \\ C_{13} & C_{23} & C_{33} & C_{34} & C_{35} & C_{36} \\ C_{14} & C_{24} & C_{34} & C_{44} & C_{45} & C_{46} \\ C_{15} & C_{25} & C_{35} & C_{45} & C_{55} & C_{56} \\ C_{16} & C_{26} & C_{36} & C_{46} & C_{56} & C_{66} \end{pmatrix} \begin{pmatrix} \epsilon_{xx} \\ \epsilon_{yy} \\ \epsilon_{zz} \\ 2\,\epsilon_{yz} \\ 2\,\epsilon_{zx} \\ 2\,\epsilon_{xy} \end{pmatrix} = \begin{pmatrix} \sigma_{xx} \\ \sigma_{yy} \\ \sigma_{zz} \\ \sigma_{yz} \\ \sigma_{zx} \\ \sigma_{xy} \end{pmatrix}, \quad \text{(S5)}$$

A total of 14 different types of strain tensors (Eq. S6) are applied to the supercell. The deformation tensor F and strain tensor E obey the relationship of Eq. S7. For every type of strain, six equidistant values of η in the range of $[-0.02, 0.02]$ are applied. The unsymmetrized elastic moduli are the linear coefficients of the LAMMPS calculated stresses and applied strains. The elastic constant calculation routine is based on the elasticity calculation guide in Materials Project.[35]

$$E_1 = \begin{pmatrix} \eta & 0 & 0 \\ 0 & 0 & 0 \\ 0 & 0 & 0 \end{pmatrix} \quad E_2 = \begin{pmatrix} 0 & 0 & 0 \\ 0 & \eta & 0 \\ 0 & 0 & 0 \end{pmatrix} \quad E_3 = \begin{pmatrix} 0 & 0 & 0 \\ 0 & 0 & 0 \\ 0 & 0 & \eta \end{pmatrix}$$
$$E_4 = \begin{pmatrix} 0 & 0 & 0 \\ 0 & 0 & \eta \\ 0 & \eta & 0 \end{pmatrix} \quad E_5 = \begin{pmatrix} 0 & 0 & \eta \\ 0 & 0 & 0 \\ \eta & 0 & 0 \end{pmatrix} \quad E_6 = \begin{pmatrix} 0 & \eta & 0 \\ \eta & 0 & 0 \\ 0 & 0 & 0 \end{pmatrix} \quad \text{(S6)}$$
$$E_7 = \begin{pmatrix} \eta & 0 & 0 \\ 0 & \eta & 0 \\ 0 & 0 & 0 \end{pmatrix} \quad E_8 = \begin{pmatrix} \eta & 0 & 0 \\ 0 & 0 & 0 \\ 0 & 0 & \eta \end{pmatrix} \quad E_9 = \begin{pmatrix} \eta & 0 & 0 \\ 0 & 0 & \eta \\ 0 & \eta & 0 \end{pmatrix}$$
$$E_{10} = \begin{pmatrix} \eta & 0 & \eta \\ 0 & 0 & 0 \\ \eta & 0 & 0 \end{pmatrix} \quad E_{11} = \begin{pmatrix} 0 & 0 & 0 \\ 0 & \eta & 0 \\ 0 & 0 & \eta \end{pmatrix} \quad E_{12} = \begin{pmatrix} 0 & 0 & \eta \\ 0 & 0 & \eta \\ \eta & \eta & 0 \end{pmatrix}$$
$$E_{13} = \begin{pmatrix} 0 & \eta & 0 \\ \eta & 0 & \eta \\ 0 & \eta & 0 \end{pmatrix} \quad E_{14} = \begin{pmatrix} 0 & \eta & \eta \\ \eta & 0 & 0 \\ \eta & 0 & 0 \end{pmatrix},$$

$$E = \frac{1}{2}\left(F^T F - 1\right), \quad \text{(S7)}$$

For bare MOF-688(one-fold), the stress was obtained by optimizing each strained structure at 0 K using the Polak-Ribiere version of the conjugate gradient (CG) algorithm, with a force tolerance of 1.0×10^{-8} kcal mol^{-1} Å^{-1}. The elastic constants of bare MOF-688(Mn) were then estimated by multiplying those of bare MOF-688(one-fold) by three with the assumption that the interactions among the three interpenetrating frameworks cause negligible effects on the total elastic constants. For the PC solvated materials, the simulation box was first equilibrated for 0.5 ns under the canonical ensemble (NVT) under Nose-Hoover thermostats with a time constant of 1 ps at 298 K, and the stress was obtained by averaging over 1.5 ns of production run under the same thermostats. The reported elastic constants were symmetrized according to the symmetry of the material (4/m point group, see Section 2). For 4/m point group, there are 7 independent elastic constants in total. The obtained values are list in the following table.

Table S5. Calculated elastic constants of MOF-688(Mn) and MOF-688(one-fold) before and after the addition of PC.

Components	MOF-688(Mn) w/o PC (GPa)	MOF-688(Mn) w/ PC (GPa)	MOF-688(one-fold) w/o PC (GPa)	MOF-688(one-fold) w/ PC (GPa)
C_{11}	5.6	8.6	1.9	2.4
C_{12}	6.0	6.8	2.0	2.4
C_{13}	1.7	6.4	0.6	2.6
C_{16}	-0.2	0.3	-0.1	-0.1
C_{33}	1.1	4.0	0.4	2.1
C_{44}	0.0	0.6	0.0	0.0
C_{66}	0.0	0.9	0.0	0.1

The bulk moduli (K) of MOF-688(Mn) and MOF-688(one-fold) were both estimated from the elastic constants using the Voigt average:[36]

$$9K_V = (C_{11} + C_{22} + C_{33}) + 2(C_{13} + C_{23} + C_{31}), \quad \text{(S8)}$$

The obtained bulk moduli are listed in the following table.

Table S6. Calculated bulk moduli (Voigt average) of MOF-688(Mn) and MOF-688(one-fold) before and after the addition of PC.

Bulk moduli	MOF-688(Mn) w/o PC (GPa)	MOF-688(Mn) w/ PC (GPa)	MOF-688(one-fold) w/o PC (GPa)	MOF-688(one-fold) w/ PC (GPa)
K_V	3.3	6.7	1.1	2.4

Section 8. Ionic conductivity as a function of temperature

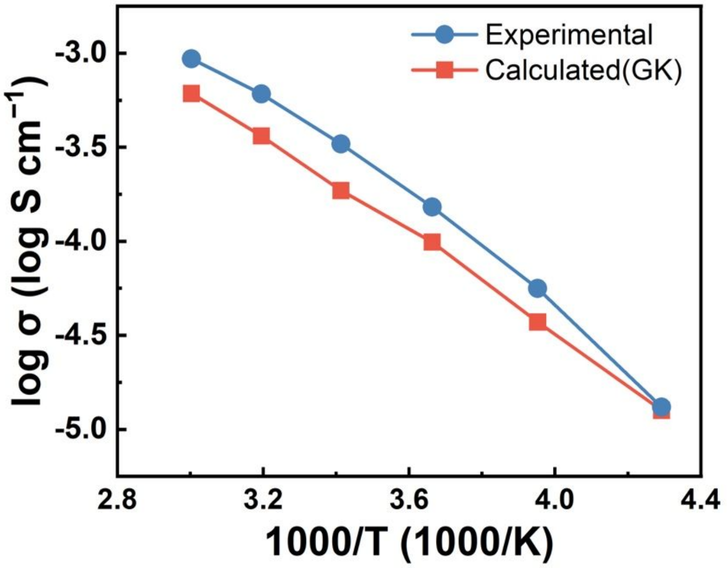

Figure S12. The calculated GK and experimentally measured conductivity of MOF-688(Mn) as a function of temperature.

The GK conductivity was obtained by analyzing the MD simulation trajectories of MOF-688(Mn) at 233, 253, 273, 293, 313, and 333 K.

Section 9. Electrostatic potential (ESP) surface of $AlMo_6$ cluster

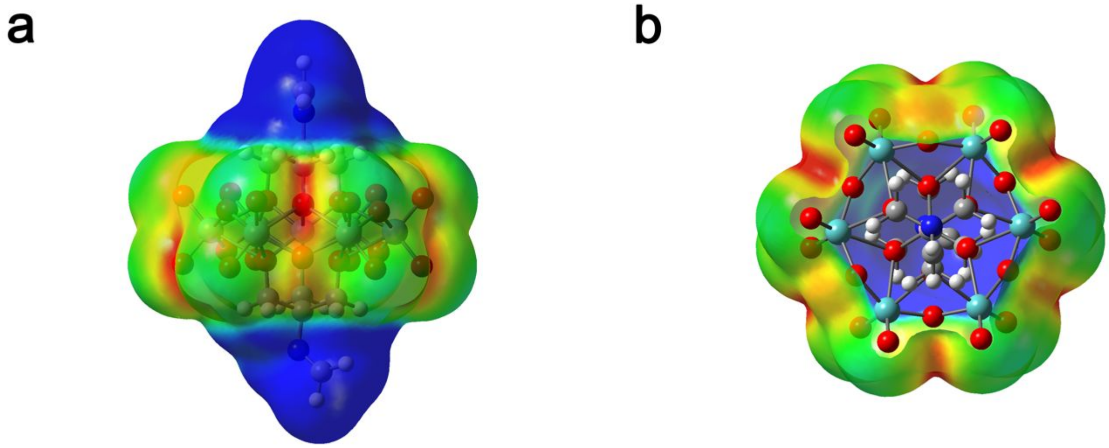

Figure S13. (a) Front and (b) top view of the electrostatic potential (ESP) surface of the $AlMo_6$ cluster. The plot shares the same color scheme as in main text, Figure 2.

Section 10. Concentration analysis

The apparent concentration ($c_{apparent}$) is defined as the amount of Li^+ (in moles) in the simulation box divided by the volume of the box V:

$$c_{apparent} = \frac{n_{Li^+}}{V}. \quad (S9)$$

The effective concentration ($c_{effective}$) is determined by mapping (with linear interpolation) the molar ratio of Li:PC to the concentration in a conventional liquid $LiPF_6$/PC electrolyte according to Self et al.[37]

References

(1) Ai, H.; Wang, Y.; Li, B.; Wu, L., Synthesis and Characterization of Single‐Side Organically Grafted Anderson‐Type Polyoxometalates. *Eur. J. Inorg. Chem.* **2014,** *2014*, 2766-2772.

(2) Zhang, J.; Luo, J.; Wang, P.; Ding, B.; Huang, Y.; Zhao, Z.; Zhang, J.; Wei, Y., Step-by-step strategy from achiral precursors to polyoxometalates-based chiral organic-inorganic hybrids. *Inorg. Chem.* **2015,** *54*, 2551-9.

(3) Xu, W.; Pei, X.; Diercks, C. S.; Lyu, H.; Ji, Z.; Yaghi, O. M., A Metal-Organic Framework of Organic Vertices and Polyoxometalate Linkers as a Solid-State Electrolyte. *J. Am. Chem. Soc.* **2019,** *141*, 17522-17526.

(4) *APEX3*, Version 8.38; Bruker-AXS: Madison, WI, 2018.

(5) *SADABS*, Version 2014/4; Bruker-AXS: Madison, WI, 2014.

(6) Dolomanov, O. V.; Bourhis, L. J.; Gildea, R. J.; Howard, J. A. K.; Puschmann, H., OLEX2: a complete structure solution, refinement and analysis program. *J. Appl. Crystallogr.* **2009,** *42*, 339-341.

(7) Sheldrick, G. M., A short history of SHELX. *Acta Cryst. A* **2008,** *64*, 112-22.

(8) Sheldrick, G. M., Crystal structure refinement with SHELXL. *Acta Cryst. C* **2015,** *71*, 3-8.

(9) Rappe, A. K.; Casewit, C. J.; Colwell, K. S.; Goddard, W. A.; Skiff, W. M., Uff, a Full Periodic-Table Force-Field for Molecular Mechanics and Molecular-Dynamics Simulations. *J. Am. Chem. Soc.* **1992,** *114*, 10024-10035.

(10) Boyd, P. G.; Moosavi, S. M.; Witman, M.; Smit, B., Force-Field Prediction of Materials Properties in Metal-Organic Frameworks. *J. Phys. Chem. Lett.* **2017,** *8*, 357-363.

(11) Jorgensen, W. L.; Maxwell, D. S.; Tirado-Rives, J., Development and Testing of the OPLS All-Atom Force Field on Conformational Energetics and Properties of Organic Liquids. *J. Am. Chem. Soc.* **1996,** *118*, 11225-11236.

(12) Kaminski, G. A.; Friesner, R. A.; Tirado-Rives, J.; Jorgensen, W. L., Evaluation and Reparametrization of the OPLS-AA Force Field for Proteins via Comparison with Accurate Quantum Chemical Calculations on Peptides†. *J. Phys. Chem. B* **2001,** *105*, 6474-6487.

(13) Jensen, K. P.; Jorgensen, W. L., Halide, Ammonium, and Alkali Metal Ion Parameters for Modeling Aqueous Solutions. *J. Chem. Theory. Comput.* **2006,** *2*, 1499-509.

(14) Bayly, C. I.; Cieplak, P.; Cornell, W. D.; Kollman, P. A., A Well-Behaved Electrostatic Potential Based Method Using Charge Restraints for Deriving Atomic Charges - the Resp Model. *J. Phys. Chem.* **1993,** *97*, 10269-10280.

(15) Rajput, N. N.; Murugesan, V.; Shin, Y.; Han, K. S.; Lau, K. C.; Chen, J.; Liu, J.; Curtiss, L. A.; Mueller, K. T.; Persson, K. A., Elucidating the Solvation Structure and Dynamics of Lithium Polysulfides Resulting from Competitive Salt and Solvent Interactions. *Chem. Mater.* **2017,** *29*, 3375-3379.

(16) Becke, A. D., Density‐functional thermochemistry. III. The role of exact exchange. *J. Chem. Phys.* **1993,** *98*, 5648-5652.

(17) Woon, D. E.; Dunning, T. H., Gaussian basis sets for use in correlated molecular calculations. III. The atoms aluminum through argon. *J. Chem. Phys.* **1993,** *98*, 1358-1371.
(18) Kendall, R. A.; Dunning, T. H.; Harrison, R. J., Electron affinities of the first - row atoms revisited. Systematic basis sets and wave functions. *J. Chem. Phys.* **1992,** *96*, 6796-6806.
(19) Hay, P. J.; Wadt, W. R., Ab initio effective core potentials for molecular calculations. Potentials for K to Au including the outermost core orbitals. *J. Chem. Phys.* **1985,** *82*, 299-310.
(20) Liu, H.; Maginn, E., A molecular dynamics investigation of the structural and dynamic properties of the ionic liquid 1-n-butyl-3-methylimidazolium bis(trifluoromethanesulfonyl)imide. *J. Chem. Phys.* **2011,** *135*, 124507.
(21) Leontyev, I.; Stuchebrukhov, A., Accounting for electronic polarization in non-polarizable force fields. *Phys. Chem. Chem. Phys.* **2011,** *13*, 2613-2626.
(22) Chaban, V., Polarizability versus mobility: atomistic force field for ionic liquids. *Phys. Chem. Chem. Phys.* **2011,** *13*, 16055-16062.
(23) Frenkel, D.; Smit, B., *Understanding Molecular Simulation: From Algorithms to Applications*, Elsevier, 2001; Vol. 1.
(24) Plimpton, S., Fast Parallel Algorithms for Short-Range Molecular-Dynamics. *J. Comput. Phys.* **1995,** *117*, 1-19.
(25) Frisch, M. J.; Trucks, G. W.; Schlegel, H. B.; Scuseria, G. E.; Robb, M. A.; Cheeseman, J. R.; Scalmani, G.; Barone, V.; Petersson, G. A.; Nakatsuji, H.; Li, X.; Caricato, M.; Marenich, A. V.; Bloino, J.; Janesko, B. G.; Gomperts, R.; Mennucci, B.; Hratchian, H. P.; Ortiz, J. V.; Izmaylov, A. F.; Sonnenberg, J. L.; Williams; Ding, F.; Lipparini, F.; Egidi, F.; Goings, J.; Peng, B.; Petrone, A.; Henderson, T.; Ranasinghe, D.; Zakrzewski, V. G.; Gao, J.; Rega, N.; Zheng, G.; Liang, W.; Hada, M.; Ehara, M.; Toyota, K.; Fukuda, R.; Hasegawa, J.; Ishida, M.; Nakajima, T.; Honda, Y.; Kitao, O.; Nakai, H.; Vreven, T.; Throssell, K.; Montgomery Jr., J. A.; Peralta, J. E.; Ogliaro, F.; Bearpark, M. J.; Heyd, J. J.; Brothers, E. N.; Kudin, K. N.; Staroverov, V. N.; Keith, T. A.; Kobayashi, R.; Normand, J.; Raghavachari, K.; Rendell, A. P.; Burant, J. C.; Iyengar, S. S.; Tomasi, J.; Cossi, M.; Millam, J. M.; Klene, M.; Adamo, C.; Cammi, R.; Ochterski, J. W.; Martin, R. L.; Morokuma, K.; Farkas, O.; Foresman, J. B.; Fox, D. J. *Gaussian 16 Rev. A.03*, Wallingford, CT, 2016.
(26) Michaud-Agrawal, N.; Denning, E. J.; Woolf, T. B.; Beckstein, O., MDAnalysis: a toolkit for the analysis of molecular dynamics simulations. *J. Comput. Chem.* **2011,** *32*, 2319-27.
(27) Hou, T.; Fong, K. D.; Wang, J.; Persson, K. A., The solvation structure, transport properties and reduction behavior of carbonate-based electrolytes of lithium-ion batteries. *Chem. Sci.* **2021,** *12*, 14740-14751.
(28) Wolfram Research, I. *Mathematica*, Wolfram Research, Inc.: Champaign, Illinois, 2021.
(29) Einstein, A., The motion of elements suspended in static liquids as claimed in the molecular kinetic theory of heat. *Ann. Phys.* **1905,** *17*, 549-560.
(30) Kulasinski, K.; Guyer, R. A., Quantification of Nanopore Networks: Application to Amorphous Polymers. *J. Phys. Chem. C* **2016,** *120*, 28144-28151.

(31) Maekawa, H.; Fujimaki, Y.; Shen, H.; Kawamura, J.; Yamamura, T., Mesopore size dependence of the ionic diffusivity in alumina based composite lithium ionic conductors. *Solid State Ion.* **2006,** *177*, 2711-2714.
(32) Fong, K. D.; Self, J.; McCloskey, B. D.; Persson, K. A., Onsager Transport Coefficients and Transference Numbers in Polyelectrolyte Solutions and Polymerized Ionic Liquids. *Macromolecules* **2020,** *53*, 9503-9512.
(33) Fong, K. D.; Self, J.; Diederichsen, K. M.; Wood, B. M.; McCloskey, B. D.; Persson, K. A., Ion Transport and the True Transference Number in Nonaqueous Polyelectrolyte Solutions for Lithium Ion Batteries. *ACS Cent Sci* **2019,** *5*, 1250-1260.
(34) Fong, K. D.; Self, J.; McCloskey, B. D.; Persson, K. A., Ion Correlations and Their Impact on Transport in Polymer-Based Electrolytes. *Macromolecules* **2021,** *54*, 2575-2591.
(35) Paier, J.; Hirschl, R.; Marsman, M.; Kresse, G., The Perdew-Burke-Ernzerhof exchange-correlation functional applied to the G2-1 test set using a plane-wave basis set. *J. Chem. Phys.* **2005,** *122*, 234102.
(36) Voight, W., Lehrbuch der Kristallphysik. *Teubner, Leipzig* **1928**.
(37) Self, J.; Fong, K. D.; Persson, K. A., Transport in Superconcentrated LiPF6 and LiBF4/Propylene Carbonate Electrolytes. *ACS Energy Lett.* **2019,** *4*, 2843-2849.
